# Hydrogen-Induced Reduction Improves the Photoelectrocatalytic Performance of Titania

**DOI:** 10.1021/acsaem.3c02707

**Published:** 2024-02-20

**Authors:** Carlos Sánchez-Sánchez, Roberto Muñoz, Elena Alfonso-González, Mariam Barawi, José I. Martínez, Elena López-Elvira, Gabriel Sánchez-Santolino, Naoya Shibata, Yuichi Ikuhara, Gary J. Ellis, Mar García-Hernández, María Francisca López, Víctor A. de la Peña O’Shea, José A. Martín-Gago

**Affiliations:** † Instituto de Ciencia de Materiales de Madrid (ICMM), CSIC, Sor Juana Inés de la Cruz 3, 28049 Madrid, Spain; ‡ Photoactivated Processes Unit, IMDEA Energy Institute, Avda. Ramón de la Sagra, 3, Móstoles, 28935 Madrid, Spain; § Institute of Engineering Innovation, School of Engineering, University of Tokyo, Yayoi 2-11-16, Bunkyo, 113-8656 Tokyo, Japan; ∥ Polymer Physics Group, Instituto de Ciencia y Tecnología de Polímeros (ICTP), CSIC, Juan de la Cierva 3, 28006 Madrid, Spain

**Keywords:** reduced titanium dioxide, hydrogen evolution reaction, plasma, light absorption, photoelectrocatalysis, STM, XPS

## Abstract

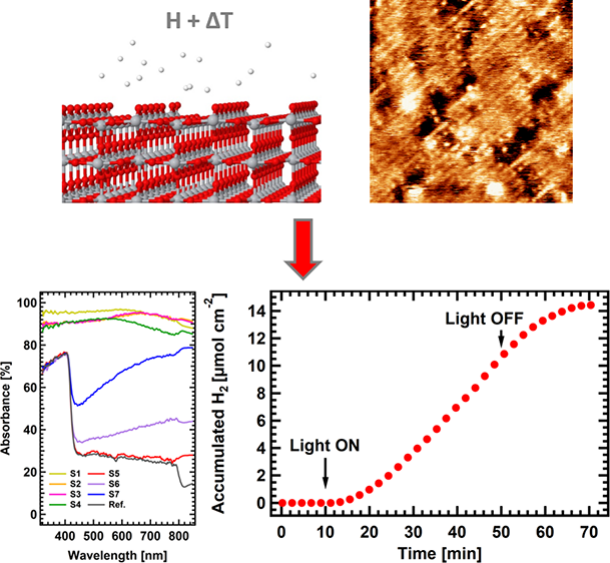

One of the main challenges to expand the use of titanium dioxide (titania) as a photocatalyst is related to its large band gap energy and the lack of an atomic scale description of the reduction mechanisms that may tailor the photocatalytic properties. We show that rutile TiO_2_ single crystals annealed in the presence of atomic hydrogen experience a strong reduction and structural rearrangement, yielding a material that exhibits enhanced light absorption, which extends from the ultraviolet to the near-infrared (NIR) spectral range, and improved photoelectrocatalytic performance. We demonstrate that both magnitudes behave oppositely: heavy/mild plasma reduction treatments lead to large/negligible spectral absorption changes and poor/enhanced (×10) photoelectrocatalytic performance, as judged from the higher photocurrent. To correlate the photoelectrochemical performance with the atomic and chemical structures of the hydrogen-reduced materials, we have modeled the process with in situ scanning tunneling microscopy measurements, which allow us to determine the initial stages of oxygen desorption and the desorption/diffusion of Ti atoms from the surface. This multiscale study opens a door toward improved materials for diverse applications such as more efficient rutile TiO_2_-based photoelectrocatalysts, green photothermal absorbers for solar energy applications, or NIR-sensing materials.

## 1 Introduction

Titanium dioxide (titania) is one of the most widely used materials in the industry, reaching a world production capacity of more than eight million metric tons in 2020, with a potential total value in the market of several billion USD.^1^ The reason for such a high consumption resides in its versatility and interesting properties such as high chemical stability, photoreactivity, UV light absorption, and biocompatibility, properties that are usually enhanced at the nanoscale, making it suitable for a plethora of industrial applications.^2^ In some of these applications, defects that appear upon TiO_2_ reduction play a pivotal role, as they constitute the active sites of the material, conferring its catalytic properties toward photoreduction of target molecules, such as water or CO_2_, for solar fuel production, or even the photodegradation of organic pollutants.^2,3^

However, one of the main challenges of TiO_2_ for its application as an efficient photocatalyst is related to its large band gap energy (∼3.1 eV) that limits the light absorption in the visible and infrared regions of the electromagnetic spectrum, which constitutes more than 90% of the total solar radiation reaching the Earth.^4,5^ Different strategies to increase titania light absorption through band gap engineering have been explored, such as doping with metallic and nonmetallic species,^6^ or generation of defects such as oxygen vacancies (O_vac_), interstitial titanium atoms (Ti_int_), hydrogenation, or disorder.^7^ In the past decade, the use of black titanium dioxide, obtained through strong hydrogenation, has been extended to improve light absorption in the visible range.^8,9^ Some pioneering works have used hydrogenation to introduce disorder or cover oxygen vacancies as a way of enhancing solar light absorption, catalytic properties,^10,11^ or solar hydrogen conversion via photoelectrochemical (PEC) water splitting.^12^ Many of these approaches involve complex alloys and metal–organic heterostructures whose combined properties are required to obtain a tuned absorption response. Thus, hydrogenation appears to provide a clean, economical, and versatile alternative to obtain materials with efficient light absorption over a broad energy range. In this direction, here we propose the use of hydrogen on rutile TiO_2_ (110) samples as a reducing agent to increase the vacancies and active sites, leading to an enhancement in the photoelectrochemical performance.

To better understand the atomic-scale interaction of atomic hydrogen with the titania surfaces, Surface Science model studies under UHV have been undertaken,^2^ providing access to the structural, chemical, and electronic properties of the surfaces. Despite the huge interest in the interaction of hydrogen with titania, the adsorption and thermal evolution of hydrogen species on rutile TiO_2_ (110) surfaces is still an open question. It has been reported that only atomic hydrogen adsorbs on the rutile TiO_2_ (110) surfaces,^13^ preferentially at O_br_ sites,^14^ where it can undergo four different thermally triggered competing processes: (i) desorption as H_2_, (ii) desorption as H_2_O, (iii) surface migration, and (iv) diffusion into the bulk to form interstitial subsurface OH groups.^14,15^ Interestingly, the reduction level of the substrate will affect hydrogen diffusion and H_2_O or H_2_ desorption.^16,17^ However, the interaction of the rutile TiO_2_ (110) surface with atomic hydrogen as a function of sample temperature has been scarcely investigated, most of the studies being focused on its desorption from initially hydrogenated surfaces close to room temperature. It is important to note that temperature can play a pivotal role in the etching mechanisms, as will be demonstrated below. Even under these constrained conditions, a possible etching effect of the surface as a consequence of H_2_O desorption has been suggested.^18^ However, a detailed and comprehensive study on the structural and electronic modification of the surfaces at the atomic level upon exposure to atomic hydrogen, as well as the correlation of the photoabsorption and photoelectrochemical performance to these changes is still missing.

In this work, the photoelectrocatalytic performance of hydrogen-exposed model single crystal rutile TiO_2_ (110) samples is evaluated and correlated with the modifications in the structural, chemical, and light absorption properties, thanks to a multitechnique approach, including an unprecedented surface science methodology. Our results indicate that the samples that exhibit the best photoelectrochemical performance are those that have undergone a superficial reduction localized at the topmost layers, while heavy reduction reaching the bulk is detrimental to their performance, in good agreement with recent results.^19,20^ Furthermore, the combination of mesoscopic and nanoscopic measurements allows rationalizing the hydrogen-induced etching mechanism, demonstrating that model studies performed on single crystalline substrates using surface science characterization techniques under highly controlled UHV conditions constitute a privileged framework to access the atomic-scale properties of the treated materials and achieve a valuable structure-performance correlation. This work will contribute to the comprehension and control of the hydrogenation process as a clean, economical, and versatile method for the development of broadband absorbers from the UV to the IR regions, efficient photocatalysts, and photothermal energy conversion devices.

## 2 Results and Discussion

**Figure 1:**
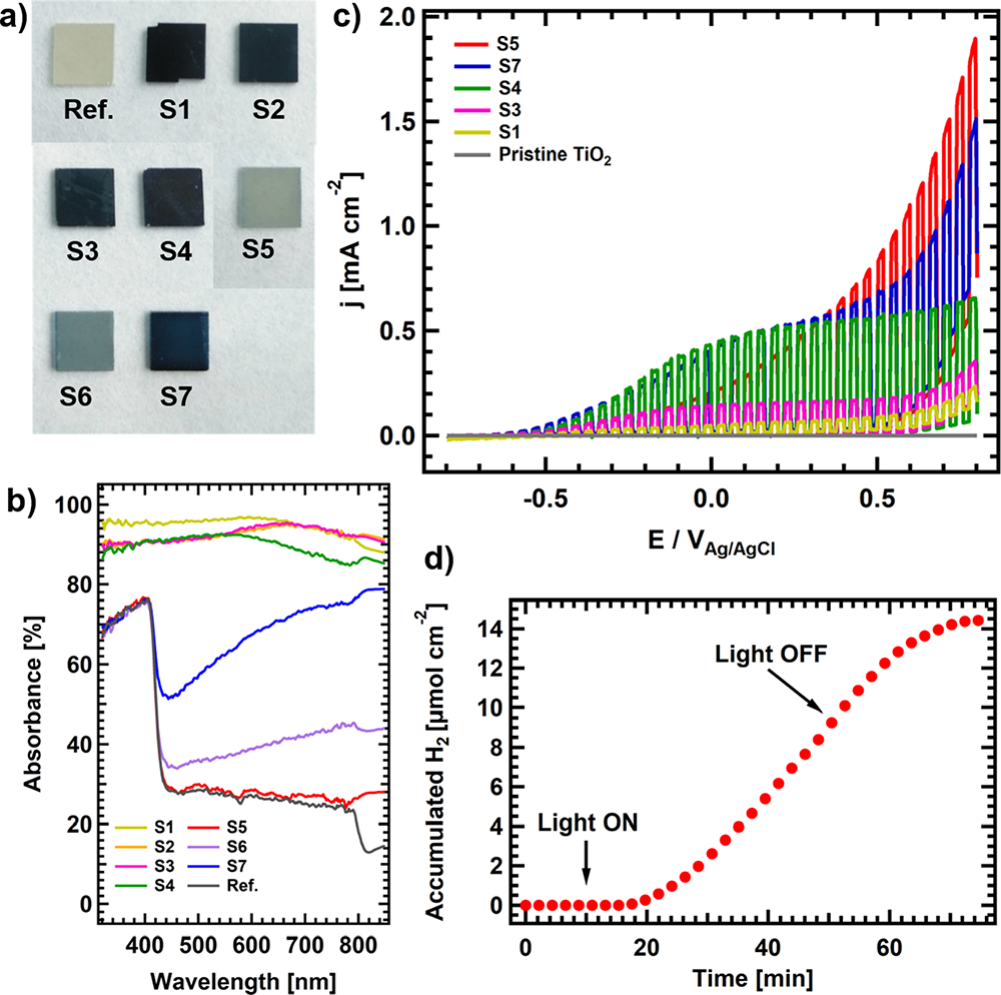
(a) Images showing the color change of the different TiO_2_ samples after each hydrogen plasma treatment. (b) Light adsorption curves in the UV**–**NIR regions for the TiO_2_ samples after different hydrogen plasma treatments. The pristine TiO_2_ sample is included as a reference. (c) LSV curves for selected treated samples covering the whole range of temperatures used in the treatments. (d) Cumulative hydrogen production vs reaction time of TiO_2_**S5** sample under solar simulated irradiation at 0.6 V (vs Ag/AgCl) during 40 min.

Rutile TiO_2_ (110) single crystals (sample **ref**. in Figure 1a) exhibit drastic color changes after hydrogen plasma etching treatments (see section 1 in ESI for more details on the plasma etching setup and process), from gray (samples **S5** and **S6**, after 30 and 60 min at 500 K in Figure 1a, respectively) to dark blue (sample **S7**, 730 K, 30 min) and black (**S4**, **S3**, **S2**, and **S1**, 950 K, 1, 30, 60, and 180 min, respectively). These changes are accompanied by an improvement in the light absorption in the visible and NIR regions, reaching an almost flat absorption above 80% from 300 to 900 nm for the treatments performed in samples **S1–S4** (Figure 1b). This absorption increase is attributed to the appearance of ingap states as a consequence of the formation of reduced Ti species that reduce the effective gap (see Figure S2). Similar band gap reduction has already been reported, for example, in ref (7). The observed modifications in the optical properties give rise to significant changes in the photoelectrochemical performance. Figure 1c shows the linear sweep voltammetry (LSV) performed on selected samples (**S1**, **S3–S5**, and **S7**) and on pristine TiO_2_ for comparison, which shows almost negligible photocurrent under simulated solar irradiation values. It is interesting to note that the sample with the mildest treatment (**S5**) presents the highest photocurrent. Contrarily, those that have undergone the more severe H-plasma treatment present a much higher light absorption in the visible/NIR regions (**S1** and **S3**) but exhibit a poor photocurrent. Finally, intermediate treatments, either for high temperature and short time (**S4**) or at moderate temperature (**S7**), show a halfway behavior, with an improved photocurrent for voltages below 0.3 V. These results are consistent with recent literature that highlights the pivotal role played by oxygen vacancies in solar energy conversion applications.^21^ While these can increase the optical absorption in the visible range, an excess can induce a metal-like behavior (degenerate semiconductor), leading to charge transfer recombination and concomitant deactivation of the photoactivity of the material. However, not only the density of vacancies is important but also their location. Surface oxygen vacancies have been reported to be beneficial to the performance of photoanodes as they improve the charge separation by narrowing the space charge layer,^22,23^ while bulk oxygen vacancies are disadvantageous, as they increase recombination dynamics and activate loss channels, with an associated decrease in photocurrent.^24^

Considering our experimental setup where platinum (almost 100% faradaic efficiency for HER) is used as a reference photocathode, we have chosen the sample with the higher photocurrent, **S5**, to be used as photoelectrode in a photoelectrochemical cell connected to a gas chromatograph to quantify the hydrogen evolution reaction (HER) produced in the counter electrode by the generated photocurrent. It must be noted that, as the HER will be directly related to the photocurrent, only this sample has been considered. In this experiment, where the reaction was carried out under conditions of 0.6 V versus Ag/AgCl, the sample is illuminated and biased during 40 min (from minute 10 to 50) and then it is let evolve, observing adequate stability (current density in the Figure S3) and yielding a production of 14 micromoles of H_2_.

**Figure 2:**
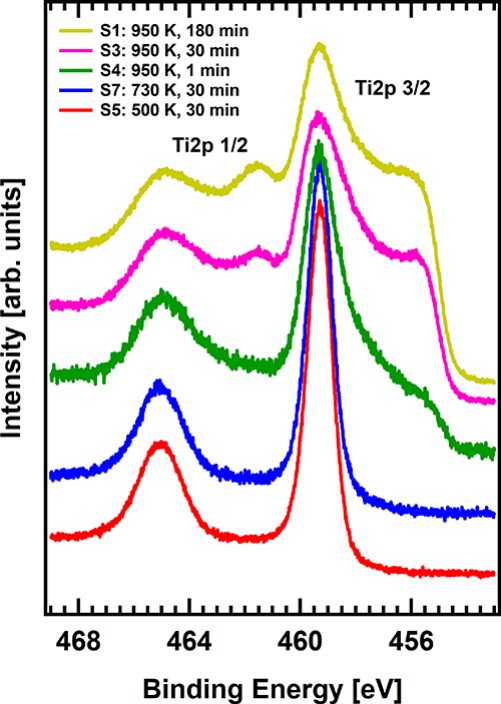
Waterfall representation of the Ti 2p core-level XPS spectra for treated samples. The *y*-axes of the spectra have been offset for clarity.

These changes in the photoelectrocatalytic behavior as a function of the reduction level can be explained on the basis of the structural, chemical, and electronic modifications of the treated samples. Figure 2 shows the XPS spectra of the Ti 2p core level for **S1**, **S3–S5**, and **S7** samples. The region below the Ti^4+^ peak (459.3 eV) is characteristic of reduced Ti species, from Ti^3+^ to Ti^2+^. Sample **S5**, presenting the higher photocurrent, shows an almost negligible amount of reduced Ti species (red curve), as indicated by the absence of any shoulder at ∼457.7 eV, which corresponds to Ti^3+^ species. This can be understood by a very superficial and subtle etching of the surface, in agreement with the light gray color exhibited by the sample (see Figure 1a). Our assumption on the formation of only superficial defects is empirically supported by the fact that, despite the very low reduction level of **S5**, XPS measurements could be carried out without any problem, i.e., no charging effects were observed, as known to occur in pristine TiO_2_ due to its large band gap. When the temperature of the sample is increased to 730 K during the etching (**S7**), the XPS spectrum starts developing a small shoulder at lower binding energies (BE) (see ESI for the complete XPS analysis including Ti 2p, C 1s, and N 1s peak deconvolution), compatible with the appearance of reduced Ti^3+^ species as a consequence of surface reduction. If the temperature is further increased up to 950 K, a much more severe reduction of the sample is observed, as judged by the development of lower BE components down to 455.4 eV. It is interesting to note that there is no evidence for the formation of Ti^1+^ and/or Ti^0^ species even after a heavy etching of the surface. Instead, the component at 455.4 eV suggests the formation of TiN and TiO_*x*_N_*y*_ species, probably as a consequence of air exposure of the plasma-etched samples (it must be noted that highly reduced Ti species are very reactive toward both N and O).^25^ Thus, the XPS results indicate the possibility to tune the reduction level of atomic species in the samples by tuning the sample temperature and duration of the plasma treatment, allowing for the control of the light absorption and amount of reduced species, which critically influence their photoelectrocatalytic performance.

The structure of the surface region seems to be crucial in determining the properties of titania. The surface roughness of the plasma-treated samples has been studied by atomic force microscopy (AFM) (see ESI, Figure S7) and it can be concluded that **S5** (soft etching, short-absorption range, and high photoelectrocatalytic performance) shows a very small RMS roughness (0.2 nm), with a value slightly higher than that observed for the pristine TiO_2_ surface (0.7 Å), in good agreement with the XPS spectrum shown above (soft etching = low reduction level = low rugosity). However, increasing the reduction temperature has a dramatic effect on the surface rugosity, that grows by a factor of ∼10. This fact indicates that the hydrogen plasma etching removes atomic species from the surface. Scanning transmission electron microscopy and electron energy loss spectroscopy (STEM-EELS) measurements (see ESI, Figure S8) of the cross-section of the **S3** sample corroborate a profound sample etching, which extends approximately 180 nm into the sample.

**Figure 3:**
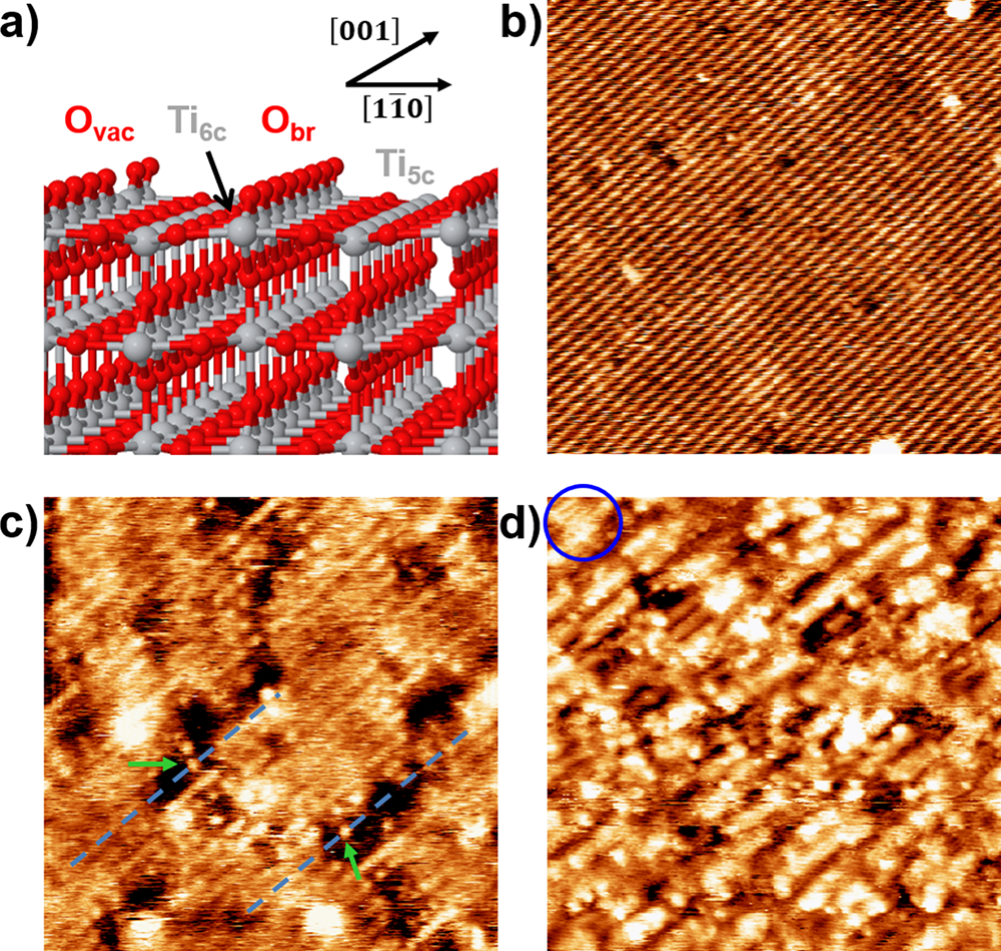
Model characterization of the H-induced etching of the TiO_2_ surface by STM. (a) Schematic representation of the rutile TiO_2_ (110)-(1 × 1) surface termination, composed of alternating rows of protruding O_br_ atoms and in-plane Ti_5c_ atoms. Red and gray atoms correspond to oxygen and titanium, respectively. (b) STM image of the clean TiO_2_ surface after several sputtering and annealing cycles under UHV conditions. Bright rows correspond to in-plane Ti_5c_ atoms.^26^ STM parameters: (25 × 25 nm), *I* = 36 pA, *V* = 1.5 V. (c,d) STM images of the TiO_2_ surface after exposure to atomic hydrogen during 2 and 30 min, respectively (substrate temperature during etching: 500 K). The blue ring in panel (d) highlights the remaining patch of the (1 × 1) surface termination. STM parameters: (15 × 15 nm), *I* = 77 pA, *V* = 1.5 V; and (35 × 35 nm), *I* = 122 pA, *V* = 1.5 V, respectively.

So far, our results present clear evidence for the possibility to tune the photoelectrochemical properties of TiO_2_ single crystals by the rational selection of the hydrogen plasma etching parameters. However, little can be said about the etching mechanism yielding this behavior and, more specifically, the surface structure of the softly etched **S5** sample, as AFM cannot produce the necessary resolution. In order to comprehend the etching mechanism at the atomic level, model UHV experiments with single crystal rutile TiO_2_ (110) samples have been carried out. In this respect, new samples were produced by exposing them to a flux of atomic and molecular hydrogen produced by a hydrogen cracker in equivalent conditions to those in the plasma procedure (see Methods section for further details). In this way, the hydrogen dose can be fine-tuned with high precision, allowing for the characterization of the initial etching stages via high-resolution STM images. In this regard, two analyses were performed: (i) analysis of the surface structure upon a variable hydrogen dose (achieved by changing the dosing time at a fixed sample temperature), and (ii) evolution of surface structure with sample temperature at a fixed dose (i.e., dosing time). Figure 3a) shows a schematic representation of the rutile TiO_2_ (110)-(1 × 1) surface. This surface is characterized by the presence of in-plane Ti and protruding O rows running along the [001] surface direction (Ti_5c_ and O_br_ rows, respectively). The corresponding STM image of the clean surface is presented in Figure 3b), where bright rows are associated with Ti_5c_ rows and not the protruding O_br_ rows due to a well-known electronic effect.^26^ Reduced (1 × 1) surfaces prepared under UHV conditions typically present two types of defects as revealed by STM, bright protrusions over the dark rows and dark depressions on the bright rows. The former is known to be due to O_br_ vacancies (O_vac_) and/or hydroxyl groups,^27^ while the origin of the latter is still not clear but could be associated with missing Ti atoms, as will be shown.

The STM images in Figure 3c,d show the evolution of two UHV-prepared rutile TiO_2_ (110)-(1 × 1) samples upon exposure to different doses of atomic hydrogen (2 and 30 min of atomic hydrogen, respectively) while being heated at 500 K. After exposure, a series of trenches appeared on the surface aligned along the [001] surface direction. At low etching times (Figure 3c), the height of the trenches corresponds to one TiO_2_ atomic layer (∼ 3.2 Å) and they extend over several unit cells along the [001] surface direction but only 1–3 unit cells in the [11̅0] direction. Interestingly, on some occasions, it was possible to distinguish individual bright dots inside the trenches (see green arrows in Figure 3c). Given their bright appearance and their location at the expected position of the Ti_5c_ rows (see dashed blue lines), these can be assigned to highly undercoordinated Ti atoms that appear as a consequence of oxygen removal in their vicinity. Their assignment to TiH species can be ruled out as it has been shown that these species are not stable at 500 K.^18^ This can be understood considering the rather low diffusion barrier (0.99 eV) to transform hydride hydrogen into hydroxyl groups.^28^

The STM image for long-term etching (30 min) shows a TiO_2_ surface completely restructured, with a corrugation of 2 versus 0.5 Å of the clean surface (see Figure S9). The surface maintains a strong directionality along the [001] surface direction. Although the surface etching is extended over the vast majority of the surface, it is still possible to observe some patches of the (1 × 1) surface structure, such as that highlighted with a blue circle.

To investigate the TiO_2_ surface reconstruction mechanism, a series of experiments modifying the atomic hydrogen dose, i.e., exposure time (1, 1.5, 2, 3, 10, and 30 min) at 500 K were performed (Figure S10). For short exposures, the creation of trenches involving both O_br_ and Ti rows was observed, while the (1 × 1) surface termination was preserved. This etching of the surface increased homogeneously with exposure time until no (1 × 1) areas were observed after 30 min (panel f).

Considering the STM and XPS results, the proposed reduction mechanism is as follows: in the first stage, atomic hydrogen is adsorbed on the O_br_ atoms of the surface giving rise to the formation of surface hydroxyl groups. After saturation of the O_br_ sites, extra hydrogen atoms will interact with the hydroxyl groups yielding H_2_O rather than adsorbing on the Ti_5c_ atoms.^29^ As a result, O_br_ atoms will desorb as H_2_O, leading to the formation of Ti^3+^ sites (either originated by the loss of O atoms or by the hydroxylation of Ti atoms), as shown by XPS. However, the STM images reveal that, given the dimensions of the trenches appearing on the surface, not only O_br_ atoms are removed but Ti atoms are also affected, probably diffusing into the bulk, occupying interstitial positions. STM simulations (Figure S11) confirm our assignation of individual bright spots inside the trenches to highly reactive undercoordinated Ti sites formed during surface reconstruction.

In addition, the existence of a non-negligible energy barrier in the process is corroborated by studying the evolution of the TiO_2_ sample with the surface temperature during the H exposure (see Figure S12). This study shows that a threshold temperature in the order of 500 K is required to initiate the H-induced etching of the surface.

**Figure 4:**
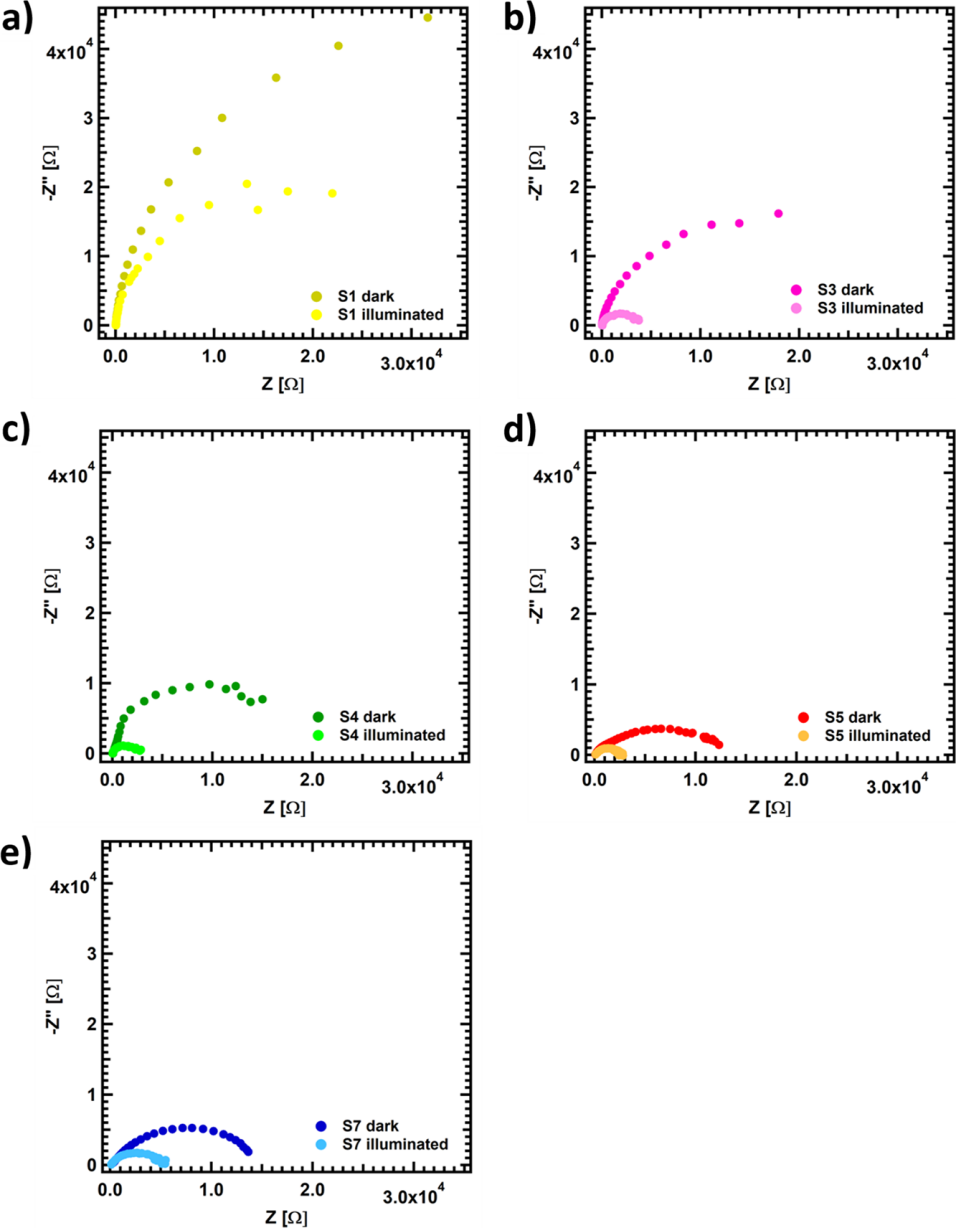
Nyquist plots obtained for **S1** (a), **S3** (b), **S4** (c), **S5** (d), and **S7** (e) at 0.4 V vs Ag/AgCl in 0.5 M Na2SO3 in dark and illumination conditions.

Finally, to establish a correlation between the etching methodology, i.e., surface restructuration and reduction with the photoelectrochemical performance of such samples, electrochemical impedance spectroscopy (EIS) measurements were performed, which show a direct correlation between surface reconstruction and the photogenerated charge transfer. Figure 4 presents Nyquist plots obtained under dark and illuminated conditions for all measured samples. Using the Randles circuit,^30,31^ the acquired semicircles can be fitted to obtain the equivalent electrical circuit composed by a series resistance *R*_S_ (that comprises the electrical contacts and electrolyte resistances) and a resistance–capacitance (*R*_CT_–*C*_CT_) in parallel (Figure S13), accounting for the TiO_2_/electrolyte interface (see Table XV in ESI). As observed, there is a strong influence of the plasma treatment on the photogenerated charge transfer. The samples treated at moderate temperatures present a more efficient charge transfer in the semiconductor-electrolyte interface. In addition, the increase in the exposure time induces an increase in *R*_CT_ and, therefore, a lower photoelectrocatalytic performance. These results are in line with the observed behavior both in the photocurrents measured and with previous literature,^32^ which pointed out that sample conductivity may also play a critical role in the photoelectrochemical performance. As observed in the photoelectrochemical measurements, the different treatments have a significant influence on the resistance associated with the TiO_2_-electrolyte charge transfer. Treatments that completely reduce TiO_2_ offer higher charge transfer resistance than milder treatments–more superficial reduction, which correlates perfectly with the PEC performance observed at the beginning of this work. Moreover, there is an acute effect when illuminating the samples in the cell with the solar simulator, decreasing the charge transfer resistance substantially in all cases. In particular, sample **S5** shows the lowest charge transfer resistance when compared to the other samples, especially under illumination conditions (see detailed resistances and capacitances obtained through the equivalent circuit in Table XV).

These results confirm the improved photoelectrochemical performance of gray titania versus extensively studied black titania, as recently reported,^21,33^ highlighting the importance of superficial versus bulk reduction.

## 3 Conclusions

We show that hydrogen plasma etching at different temperatures is an efficient methodology to selectively reduce TiO_2_, which may lead to changes in its structural, chemical, and optoelectronic properties and, as a consequence, to improved photoelectrocatalytic performance.

Severe plasma etching conditions (resulting in black titania) lead to an enhancement of light absorption from UV to NIR range and a poor photoelectrocatalytic performance. On the other hand, mild plasma etching conditions (gray titania) do not reveal important changes in the light absorption spectral range in the visible regime but show a substantial increase in the light-driven reactions.

This behavior is rationalized in terms of the significant structural and chemical changes produced on the TiO_2_ surface after the plasma-etching treatment. Low temperature and short times lead to a restructuration of the first layers of TiO_2_ by the creation of surface-confined oxygen vacancies and the emergence of highly reactive undercoordinated Ti sites and hydrogenated species. Contrastingly, the increase of the reduction temperature or exposure time results in the propagation of the structural and chemical changes from the surface to the bulk of TiO_2_, which decreases the generation and transfer of photogenerated charges. Photoelectrocatalytic reactions are positively affected by the presence of surface defects, in the form of reduced Ti species, while deeper defects in the bulk have a negative effect, possibly inducing charge recombination before reaction. It is worth noting that the proposed methodology, based on the interaction of atomic H and TiO_2_ surfaces, can have a clear impact in applications as the use of plasmas at mild temperatures is routinely used in industry, thus opening a door to improved photoelectrocatalysts, even more, if nanoparticles are considered. This study contributes to the current understanding of reduced titania as an ideal candidate for, among others, the development of TiO_2_-based light-driven devices for solar energy conversion technologies.

## 4 Experimental Section

In this work, two different but complementary types of experiments have been carried out: model UHV experiments and more technologically relevant plasma experiments. It should be noted that, in each type of experiment, specific samples have been prepared trying to use equivalent conditions. In this way, samples prepared under H-plasma conditions (**S1–S7**) have been characterized by ex-situ techniques and XPS (in this case, samples have been transferred through the air), while UHV samples have been integrally prepared and characterized under UHV conditions, without being exposed to air at any stage.

### 4.1 UHV Experiments

STM UHV experiments were undertaken in a UHV chamber equipped with an RT-STM (ScientaOmicron) at a base pressure of 1.0 × 10^–10^ mbar. Rutile TiO_2_ (110)-(1 × 1) single crystals (Mateck) were prepared by repeated sputtering (Ar^+^, 1 kV) and annealing (1100 K) cycles until observed to be clean by LEED and STM. Atomic hydrogen exposure was performed following a protocol similar to that reported elsewhere.^34^ H is produced by an H_2_ cracker (Specs) operated at 1.1 kV and 40 mA, with a hydrogen partial pressure in the chamber of 5.0 × 10^–8^ mbar (the estimated pressure at the exit of the cracker is in the 10^–4^ mbar regime). STM images were acquired with Dulcinea electronics (Nanotec) in the constant current mode and analyzed with the WSxM software.^35^

XPS measurements were performed in a UHV chamber (base pressure of 1.0 × 10^–10^ mbar) equipped with a PHOIBOS 100 1D delay line detector electron/ion analyzer and a monochromatic Al Kα anode (1486.6 eV). UHV samples were transferred via a UHV suitcase to avoid contamination, while plasma samples were transferred in the air. The binding energy (BE) scale was calibrated with respect to the Ti 2p core-level peak at 459.3 eV.^36^ All peaks shown in this work were fitted using Voigt functions after subtraction of a Shirley-type background. In all cases, the Lorentzian full width at half-maximum (fwhm-L) was kept constant during the fitting (0.35 eV for Ti 2p and O 1s) while the Gaussian fit (fwhm-G) was allowed to change. A pass energy of 15 eV was used in all cases.

### 4.2 Plasma Experiments

Remote electron cyclotron resonance chemical vapor deposition r-(ECR-CVD) plasma technique (ASTEX AX 4500 ECR) was used for the etching of TiO_2_ single crystals with hydrogen. The system consists of a microwave power source, a two-zone chamber with a plasma chamber separated from the reaction chamber, and a two-stage pumping system.^37^ The etching parameters and profile are described in the Supporting Information, Section S1.

Light absorption was measured using a SHIMADZU SolidSpec–3700 spectrophotometer equipped with an integrating sphere (BaSO_4_ reflectance standard). To obtain the light absorption (*A*) values, the transmittance (*T*) and total reflectance (*R*) were measured with the light beam perpendicular to the sample. Then, the (*A* + *T* + *R*) = 100% relation was applied. It is worth noting that the *T* values depend on the thickness of the samples (500 μm) and the final (*A*) quantitative values are related to the reflectance material.

### 4.3 AFM Experiments

AFM measurements were performed at room temperature and ambient conditions with a commercial instrument and software from Nanotec Electrónica.^33^ Dynamic operation mode was employed, exciting the tip at its resonance frequency (∼75 kHz) to acquire topographic information on the samples. Aluminum-coated silicon cantilevers (*k* = 3 N/m) were used.

### 4.4 STEM-EELS Experiments

STEM-EELS measurements were carried out in an aberration-corrected JEOL JEM-ARM300cF installed at the University of Tokyo, operated at 300 kV, and equipped with a cold field emission gun and an EELS Quantum spectrometer. For spectrum imaging, the electron beam was scanned along the region of interest, and an EEL spectrum was acquired at every pixel with an acquisition time of 2 s/pixel. The cross-sectioned specimen was prepared by conventional mechanical polishing and Ar ion milling.

### 4.5 PEC Experiments

The (photo)electrochemical measurements with pristine and reduced TiO_2_ were performed using a three-electrode cell with a quartz window, in an aqueous solution of 0.5 M Na_2_SO_3_ at pH 9. All TiO_2_ samples were used as working electrodes. The counter and reference electrodes were platinum and a Ag/AgCl wire, respectively. The electrochemical voltage and responses under dark and illumination conditions were measured with a potentiostat-galvanostat PGSTAT302N equipped with an integrated impedance module FRAII. A modulation amplitude of 10 mV was used in the frequency range from 1 to 10,000 Hz in the electrochemical impedance spectroscopy measurements (EIS). The experiments were conducted under an argon flow of 50 sccm through the top of the cell. A Solar Simulator (LOT LSH302 Xe lamp with an LSZ389 AM1.5 Global filter) was used as a light source.

To measure the reaction products, the cell was connected to a gas chromatograph (Agilent micro-GC 490) equipped with a MS5A column with a temperature of 60 °C and a TDC detector.

### 4.6 Theoretical Methods

First-principles atomistic simulations were performed to model, in a first step, the structure of a clean and a reduced rutile TiO_2_(110)-(1 × 1) surfaces, and afterward, on the basis of the established ground-state structures, to compute their corresponding theoretical Keldish-Green STM images. For this purpose, we have effectively combined the plane-wave and localized-basis-set Density Functional Theory (DFT) schemes as implemented in the QUANTUM ESPRESSO^38^ and FIREBALL^39^ simulation packages, respectively. Further information about theoretical methods and models can be found in the Supporting Information.

## Supporting Information Available

Plasma etching process and setup, estimation of the optical band gap from Kubelka–Munk analysis, HER, XPS analysis, and peak deconvolution, AFM characterization, STEM-EELS characterization, analysis of the corrugation of clean and etched surfaces under UHV conditions, evolution of the TiO_2_ surface with atomic hydrogen dose, simulated STM images of the etched surface, evolution of the TiO_2_ surface with sample temperature during atomic hydrogen dose, EIS, and theoretical methods and models (PDF)
