# Supplementary material for: Hydrogen-Induced Reduction Improves the Photoelectrocatalytic Performance of Titania
Source: ACS Appl Energy Mater. 2024 Feb 20;7(6):2101–8. doi: 10.1021/acsaem.3c02707 (PMC10966639; doi:10.1021/acsaem.3c02707)
Supplement: Supplementary file 1 — ae3c02707_si_001.pdf [file ae3c02707_si_001.pdf]

Supporting Information

Hydrogen-induced Reduction Improves the Photoelectrocatalytic Performance of Titania

Carlos Sánchez-Sánchez[1], Roberto Muñoz[1], Elena Alfonso-González [2], Mariam Barawi[2], José I. Martínez[1], Elena López-Elvira[1], Gabriel Sánchez-Santolino[1†], Naoya Shibata[3], Yuichi Ikuhara[3], Gary J. Ellis[4], Mar García-Hernández[1], María Francisca López[1], Víctor A. de la Peña O´Shea[2], José A. Martín-Gago[1*]*

[1] Instituto de Ciencia de Materiales de Madrid (ICMM), CSIC, Sor Juana Inés de la Cruz 3, 28049 Madrid (Spain).

[2] Photoactivated Processes Unit, IMDEA Energy Institute, Avda. Ramón de la Sagra, 3. 28935 Móstoles, Madrid (Spain).

[3]Institute of Engineering Innovation, School of Engineering, University of Tokyo, Yayoi 2-11-16, Bunkyo, 113-8656 Tokyo (Japan).

[4]Polymer Physics Group, Instituto de Ciencia y Tecnología de Polímeros (ICTP), CSIC, Juan de la Cierva 3, 28006 Madrid (Spain).

Email: cssanchez@icmm.csic.es; gago@icmm.csic.es

Table of Contents

1.- Plasma etching process and setup.

2.- Estimation of the optical band gap from Kubelka-Munk analysis.

3.- Hydrogen Evolution Reaction (HER).

4.- XPS analysis and peak deconvolution.

5.- AFM characterization.

6.- STEM-EELS characterization.

7.- Analysis of the corrugation of clean and etched surfaces under UHV conditions.

8.- Evolution of the TiO_2 surface with atomic hydrogen dose.

9.- Simulated STM images of the etched surface.

10.- Evolution of the TiO_2 surface with sample temperature during atomic hydrogen dose.

11.- Electrochemical Impedance Spectroscopy.

12.- Theoretical methods and models.

1.- Plasma etching process and setup

The hydrogen etching process is based on the exposure of the target sample (in our case, rutile TiO_2 (110) single crystals) to a hydrogen plasma at a certain temperature. This plasma will be formed by both atomic and molecular hydrogen species (as well as ionized species) in an approximate ratio of 1:10.[1] The former, which will interact with the atomic species on the surface (mainly oxygen atoms), will be responsible for the surface etching, reduction and hydrogenation.

Figure S1 shows a schematic diagram that explains the hydrogen plasma etching process. The whole protocol comprises three steps. In the *preliminary step*, the chamber is evacuated to a pressure of 7 x 10^{-5} mbar and subsequently filled with H_2 until a partial pressure of 5.4 x 10^{-2} mbar is reached. Then, the sample temperature is gradually increased, a step that usually takes around 40 min. Once the final temperature and H_2 pressure are achieved, we initiate the second step of the process: the reaction step. During this phase, the hot sample is exposed to a hydrogen plasma over a variable time, depending on the desired etching level. Once the desired time has been completed, the plasma is switched off and the sample is allowed to cool to RT in the H_2 atmosphere (cooling step). Finally, once the sample has reached RT, the H_2 is evacuated. This etching process was undertaken for a full set of samples using different annealing temperatures (500, 730, and 950 K) and etching times (1, 30, 60, and 180 min).

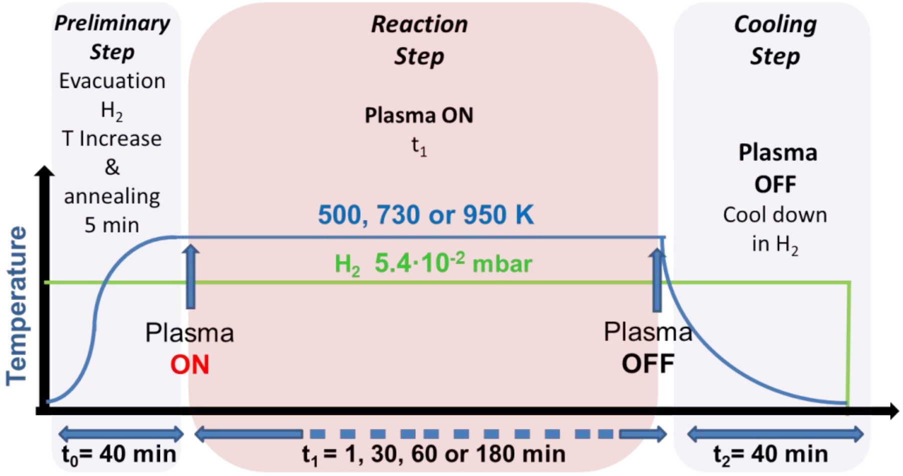

Figure S1.- Schematic representation of the plasma-etching process showing the three steps involved.

2.- Estimation of the optical band gap from Kubelka-Munk analysis.

It is possible to extract an estimation of the optical band gap of a semiconductor from diffuse reflectance measurements via a Tauc plot of the Kubelka-Munk function.[2,3] However, the value of the band gap obtained through this approach has to be considered with caution in those modified semiconductors where the absorption is due to new ingap states. As this is our case, we use this approach just to get a flavour on the effect of H-induced reduction on the gap of the material. Figure S2 shows the Tauc plots for the different samples analyzed in Figure 1b. As it can be observed, the region to be fitted is only well-defined in those samples with the lower level of reduction (**S5**-**S7**). On the contrary, those heavily reduced are difficult to fit. We have tried to get an estimate carrying out a fit in a similar energy range as that used in the other samples, although the values extracted are very speculative and with a high error.

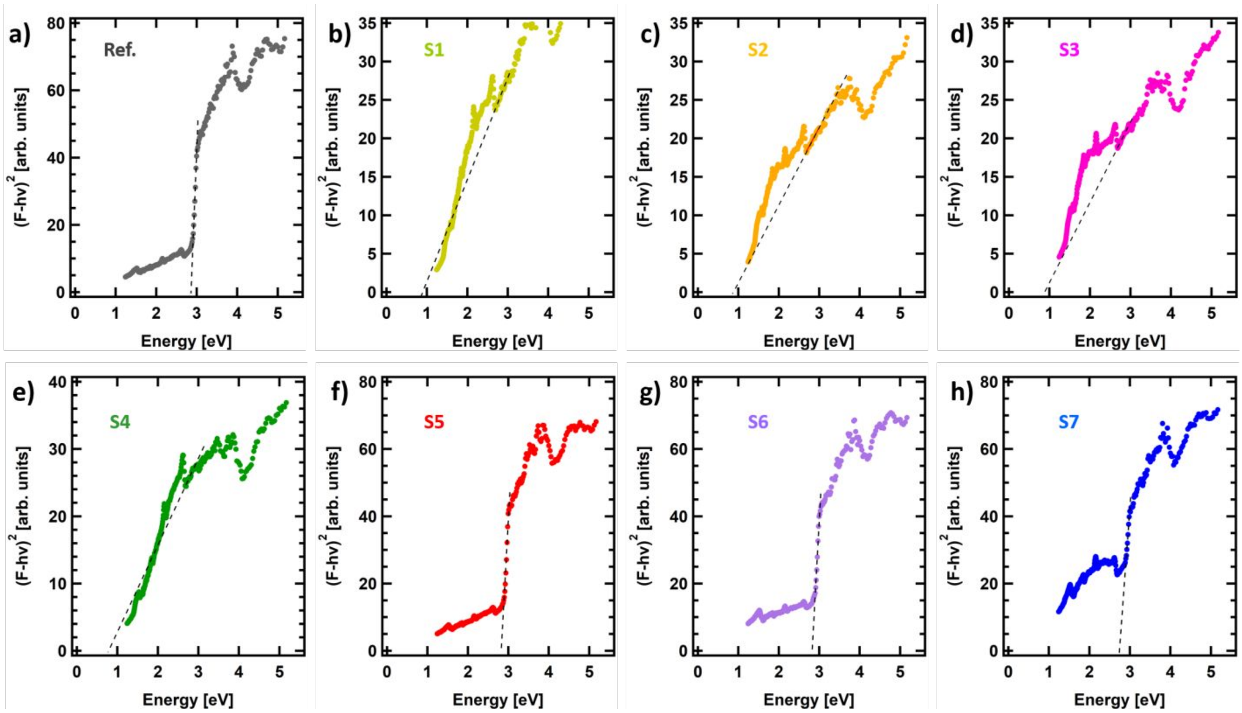

Figure S2.- Tauc plots of the Kubelka-Munk function for the different samples analyzed. a) Reference pristine TiO2, b) **S1**, c) **S2**, d) **S3**, e) **S4**, f) **S5**, g) **S6** and h) **S7**. Dashed lines denote the fit carried to estimate the band gap.

From this analysis, we extract the following band gaps:

	Ref.	S1	S2	S3	S4	S5	S6	S7
**Band gap [eV]**	2.9 ± 0.1	0.9 ± 0.3	0.8 ± 0.3	0.9 ± 0.3	0.8 ± 0.3	2.8 ± 0.1	2.8 ± 0.1	2.7 ± 0.1

Table I.- Extracted band gap values.

Thus, we can define two clear regimes: i) samples with low reduction level, i.e. mainly superficial (**S5-S7**), are slightly affected in their band gap (with band gap reductions in the order of 3 – 7 %), as already inferred from XPS results, ii) heavily reduced samples (**S1-S4**) present much lower band gaps, in all cases below 1 eV (band gap reductions in the order of 70 %).

3.- Hydrogen Evolution Reaction (HER).

To determine the stability of the etched photoelectrodes during the HER, the current density was monitored during the 40 min of illumination, showing an adequate stability that was manifested as a relatively constant photocurrent during the whole reaction (Figure S3).

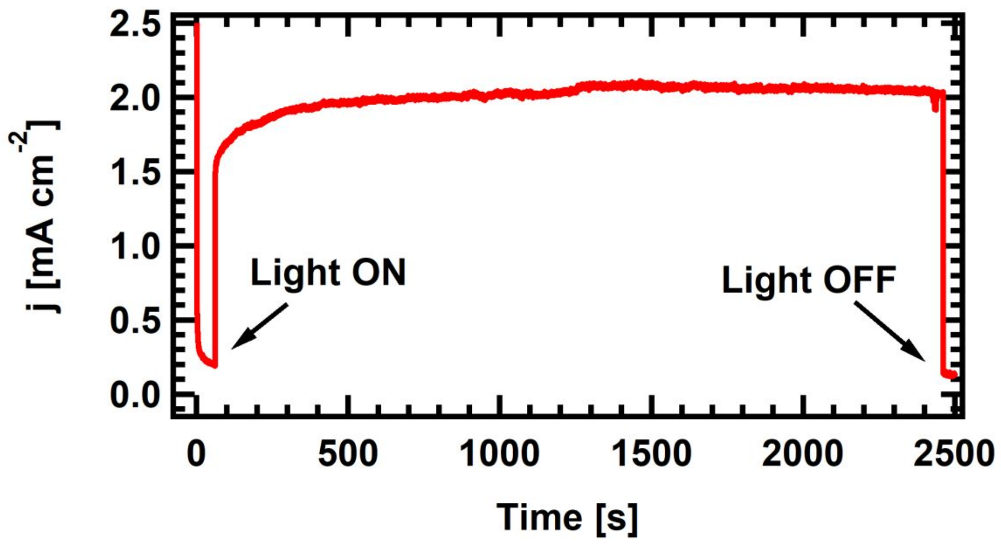

Figure S3.- Photoelectrode stability observed via the current density during a 40 min illumination.

4.- XPS analysis and peak deconvolution.

Figures S4, S5 and S6 present the deconvolution of the Ti 2p, C 1s and N 1s core level XPS spectra of the plasma-etched samples **S5**, **S7**, **S4**, **S3**, and **S1**, respectively. In the case of Ti 2p, to properly fit the spectrum for each condition, four doublets were needed, which correspond to Ti^{4+} (459.3 eV, blue curve), Ti^{3+} (~ 457.7 eV, green curve), and Ti^{2+} (~ 456.4 eV, pink curve). We associate the fourth component (~ 455.5 eV, gold curve) to TiN and TiO_xN_y species appearing upon exposure of etched samples to air due to transportation from the plasma to the UHV chamber. Thus, each oxidation state exhibits two peaks, *i.e*. a doublet, formed by the Ti $2p_{3/2}$ and $2p_{1/2}$ signals, which are separated by the Ti 2p spin-orbit splitting, 5.6 eV.

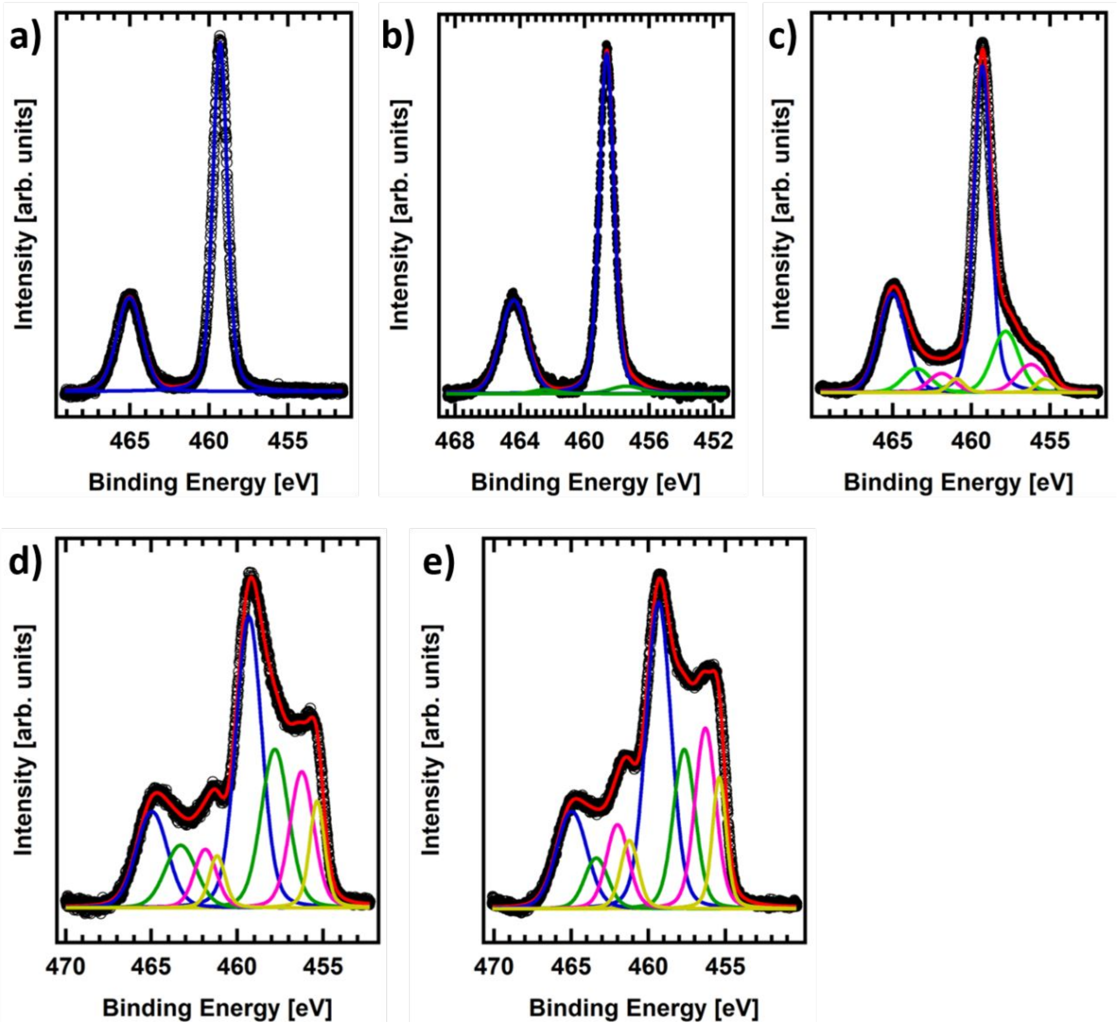

Figure S4.- XPS fits of the Ti 2p core level of the different plasma-etched samples. a) Sample **S5**, b) sample **S7**, c) sample **S4**, d) sample **S3**, and e) sample **S1**.

Table II.- Fitting parameters Ti 2p core level of **S5**

	Ti^{4+} $2p_{3/2}$	Ti^{4+} $2p_{1/2}$
**Background**	Shirley	Shirley
**BE**	459.3	465.1
**Area**	18883	8674
**FWHM-L**	0.35	0.35
**FWHM-G**	0.86	1.71

Table III.- Fitting parameters Ti 2p core level of **S7**

	Ti^{4+} $2p_{3/2}$	Ti^{3+} $2p_{3/2}$	Ti^{4+} $2p_{1/2}$
**Background**	Shirley	Shirley	Shirley
**BE**	459.3	458.1	465.0
**Area**	13493	620	6524
**FWHM-L**	0.35	0.35	0.35
**FWHM-G**	0.85	2.1	1.80

Table IV.- Fitting parameters Ti 2p core level of **S4**

	Ti^{4+} $2p_{3/2}$	Ti^{3+} $2p_{3/2}$	TiN_x $2p_{3/2}$	Ti^{4+} $2p_{1/2}$	Ti^{3+} $2p_{1/2}$	TiN_x $2p_{1/2}$
**Background**	Shirley	Shirley	Shirley	Shirley	Shirley	Shirley
**BE**	459.3	457.7	455.7	465.0	463.3	461.4
**Area**	15665	4500	2054	7204	1812	1492
**FWHM-L**	0.35	0.35	0.35	0.35	0.35	0.35
**FWHM-G**	1.08	1.78	1.51	1.80	1.80	1.63

Table V.- Fitting parameters Ti 2p core level of **S3**

	Ti^{4+} $2p_{3/2}$	Ti^{3+} $2p_{3/2}$	Ti^{2+} $2p_{3/2}$	TiN_x $2p_{3/2}$	Ti^{4+} $2p_{1/2}$	Ti^{3+} $2p_{1/2}$	Ti^{2+} $2p_{1/2}$	TiN_x $2p_{1/2}$
**Background**	Shirley	Shirley	Shirley	Shirley	Shirley	Shirley	Shirley	Shirley
**BE**	459.3	457.9	456.6	455.5	465.0	463.6	462.2	461.0
**Area**	10294	7347	2750	5447	3816	2930	1788	2698
**FWHM-L**	0.35	0.35	0.35	0.35	0.35	0.35	0.35	0.35
**FWHM-G**	1.46	1.70	1.03	1.18	1.81	1.80	1.36	1.20

Table VI.- Fitting parameters Ti 2p core level of **S1**

	Ti^{4+} $2p_{3/2}$	Ti^{3+} $2p_{3/2}$	Ti^{2+} $2p_{3/2}$	TiN_x $2p_{3/2}$	Ti^{4+} $2p_{1/2}$	Ti^{3+} $2p_{1/2}$	Ti^{2+} $2p_{1/2}$	TiN_x $2p_{1/2}$
**Background**	Shirley	Shirley	Shirley	Shirley	Shirley	Shirley	Shirley	Shirley
**BE**	459.3	457.6	456.2	455.3	465.0	463.5	462.1	461.2
**Area**	7624	4586	3420	2100	2879	1243	1919	1830
**FWHM-L**	0.35	0.35	0.35	0.35	0.35	0.35	0.35	0.35
**FWHM-G**	1.65	1.70	1.30	0.86	2.08	1.65	1.70	1.32

In the case of C 1s, two to four components where necessary to fit the curve. The two common peaks appear at around 286 and 290 eV, which are associated to C-O and C=O contaminants as a consequence of carbon interaction with the surface oxygen. For heavier reduction levels (**S1**, **S3** and **S4**), a new component develops at 284.8 eV, which is characteristic of adventitious carbon. Finally, for the heavily reduced **S1** sample, a fourth component at 282.5 eV appears. This component has been assigned to carbide species with Ti atoms from the sample.[4] This result corroborates the high reduction of such samples where Ti atoms become available to interact with the surrounding media when exposed to air.

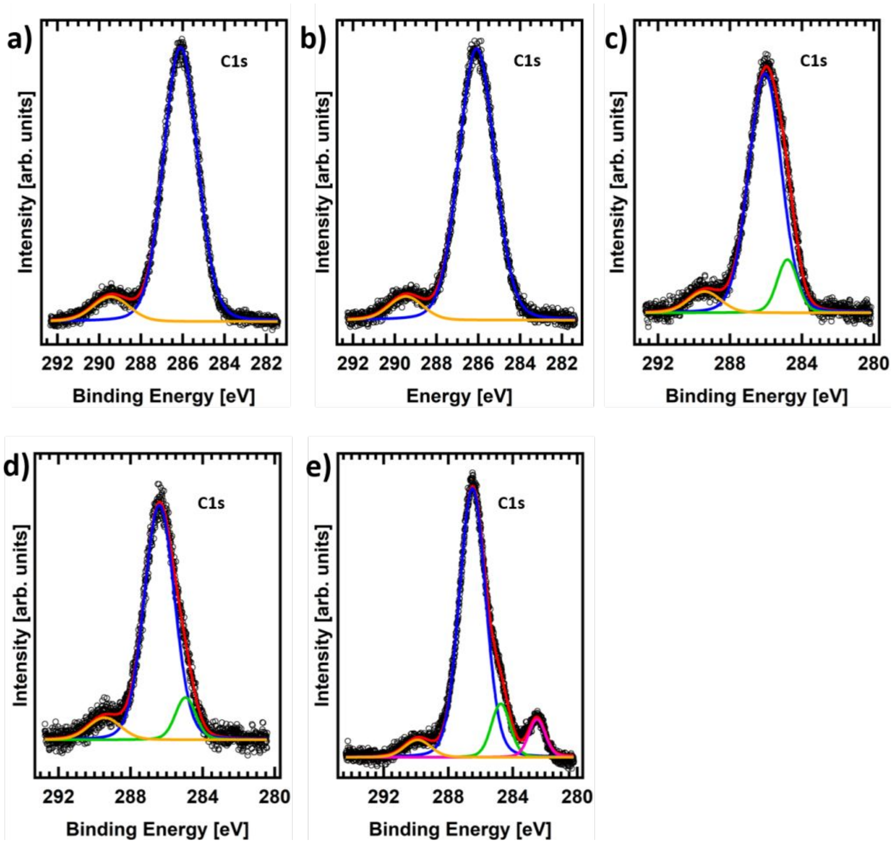

Figure S5.- XPS fits of the C 1s core level of the different plasma-etched samples. a) Sample **S5**, b) sample **S7**, c) sample **S4**, d) sample **S3**, and e) sample **S1**.

Table VII.- Fitting parameters C 1s core level of **S5**

	C-O	C=O
**Background**	Shirley	Shirley
**BE**	286.1	289.4
**Area**	10215	987
**FWHM-L**	0.25	0.25
**FWHM-G**	1.78	1.93

Table VIII.- Fitting parameters C 1s core level of **S7**

	C-O	C=O
**Background**	Shirley	Shirley
**BE**	286.1	289.5
**Area**	9792	819
**FWHM-L**	0.25	0.25
**FWHM-G**	1.88	1.79

Table IX.- Fitting parameters C 1s core level of **S4**

	Advent. C	C-O	C=O
**Background**	Shirley	Shirley	Shirley
**BE**	284.8	286.0	289.4
**Area**	314	2192	195
**FWHM-L**	0.25	0.25	0.25
**FWHM-G**	1.20	2.0	2.0

Table X.- Fitting parameters C 1s core level of **S3**

	Advent. C	C-O	C=O
**Background**	Shirley	Shirley	Shirley
**BE**	284.9	286.4	289.4
**Area**	300	2428	243
**FWHM-L**	0.25	0.25	0.25
**FWHM-G**	1.19	1.85	1.99

Table XI.- Fitting parameters C 1s core level of **S1**

	TiC_x	Advent. C	C-O	C=O
**Background**	Shirley	Shirley	Shirley	Shirley
**BE**	282.5	284.7	286.5	289.9
**Area**	181.1	284.1	1942.5	129.6
**FWHM-L**	0.25	0.25	0.25	0.25
**FWHM-G**	1.09	1.24	1.78	1.8

Finally, the N 1s core level emissions can be fitted with up to three components, once more depending on the reduction level of the samples. For those mildly reduced (**S5** and **S7**), no nitrogen is observed in our experimental setup. Just for **S7** a component at around 397 eV seems

to start to develop. These results indicate that superficial, mild reduction of the surface is not enough to form nitrogen-based species on the surface. However, when the reduction is substantially increased (**S1, S3** and **S4**), three components appear at around 397.2, 396.5 and 399 eV. The former two are assigned to different TiN_x species as a consequence of the interaction of interstitial Ti, formed upon reduction, with atmospheric N, while the one at higher BE is assigned to TiN_xO_y species, as indicated in the main text.

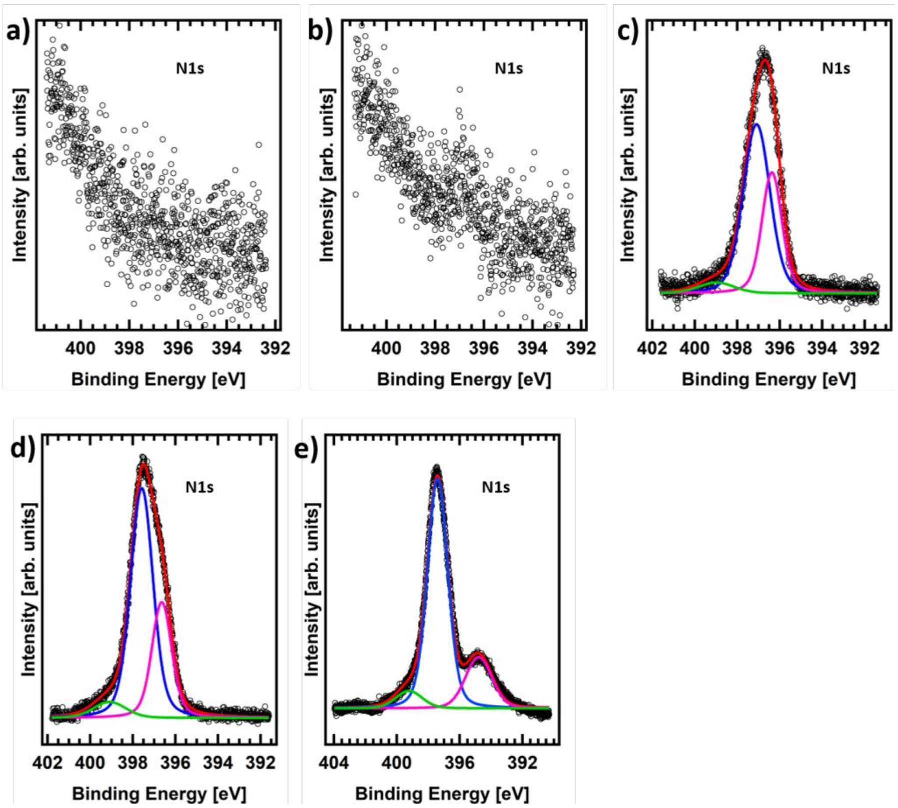

Figure S6.- XPS fits of the N 1s core level of the different plasma-etched samples. a) Sample **S5**, b) sample **S7**, c) sample **S4**, d) sample **S3**, and e) sample **S1**.

Table XII.- Fitting parameters N 1s core level of **S4**

	TiN_x	TiN_y	TiN_xO_y
**Background**	Shirley	Shirley	Shirley
**BE**	396.35	397.1	399.0
**Area**	516	941	78
**FWHM-L**	0.35	0.35	0.35
**FWHM-G**	0.90	1.30	1.80

Table XIII.- Fitting parameters N 1s core level of **S3**

	TiN_x	TiN_y	TiN_xO_y
**Background**	Shirley	Shirley	Shirley
**BE**	396.6	397.6	399.1
**Area**	1440	3199	310
**FWHM-L**	0.35	0.35	0.35
**FWHM-G**	0.89	1.05	1.61

Table XIV.- Fitting parameters N 1s core level of **S1**

	TiN_x	TiN_y	TiN_xO_y
**Background**	Shirley	Shirley	Shirley
**BE**	394.8	397.4	399.2
**Area**	566	2000	192
**FWHM-L**	0.35	0.35	0.35
**FWHM-G**	1.8	1.34	1.8

5.- AFM characterization

The surface roughness (RMS) determined by *ex-situ* AFM shows important differences after plasma treatment between selected **S5**, **S7**, and **S3** samples (Figures S7 a-d). **S5** presents an RMS roughness value of approximately 0.2 nm, indicating a very low etching of the surface, slightly higher than that observed for the pristine TiO_2 surface (0.7 Å), in good agreement with the XPS spectrum shown above. However, increasing the reduction temperature has a dramatic effect on the surface rugosity, growing by a factor of ~10 for each superior temperature, to 2.3 nm for **S7** (730 K) and 23 nm for **S3** (950 K). Thus, there is a clear relation between superficial restructuration induced by the plasma etching and the appearance of reduced Ti species in XPS, indicating that the hydrogen plasma etching, which includes atomic and molecular hydrogen, removes atomic species from the surface.

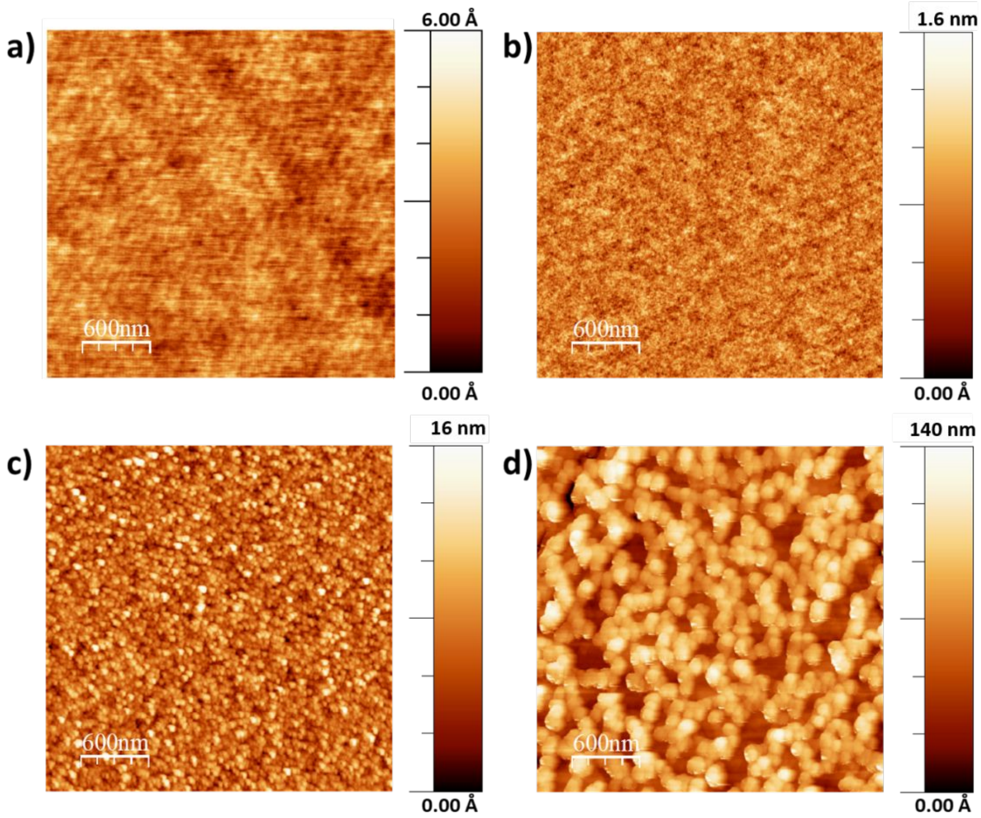

Figure S7.- Topography AFM images of the rutile TiO_2 (110) samples after different plasma etching processes. **a)** Pristine TiO_2 sample, as reference. **b) S5**: 500 K, 30 min. **c) S7**: 730 K, 30 min. **d) S3**: 950 K, 30 min.

6.- STEM-EELS characterization

STEM-EELS measurements of the cross-section of highly reduced samples (**S3**, 950 K, 30 min) corroborate this deep sample etching, as observed in Figure S8a, where the etching extends approximately 180 nm into the sample. Furthermore, it is possible to observe bright rows that run along the [001] surface direction (yellow arrows), indicating a preferential etching direction. As shown below, this result is in excellent agreement with the model STM characterization carried out, where the formation of linear structures along this direction is observed. In addition, atomic distribution concentration, acquired in an EELS line-scan taken along the surface normal (green line in Figure S8a), shows that around 150 nm the Ti/O ratio starts increasing as the scan approaches the surface (Figure S8b). This variation is due to the abovementioned removal of oxygen atoms by the hydrogen plasma and the diffusion of Ti atoms, indicating a strong

perturbation of the bulk stoichiometry under severe annealing treatment. This is more evident at the topmost layers, where an inversion in the concentration of O and Ti occurs (see the brighter line marked by the red arrow in both panels of Figure S8). As it is well known, defects in the form of reduced Ti species as a consequence of surface reduction and restructuration yields the appearance of new in-gap states and a subsequent progressive decrease of the bandgap,[5] which can explain the increase of the light absorption in the visible and near-infrared regions, as shown in Figure 1.

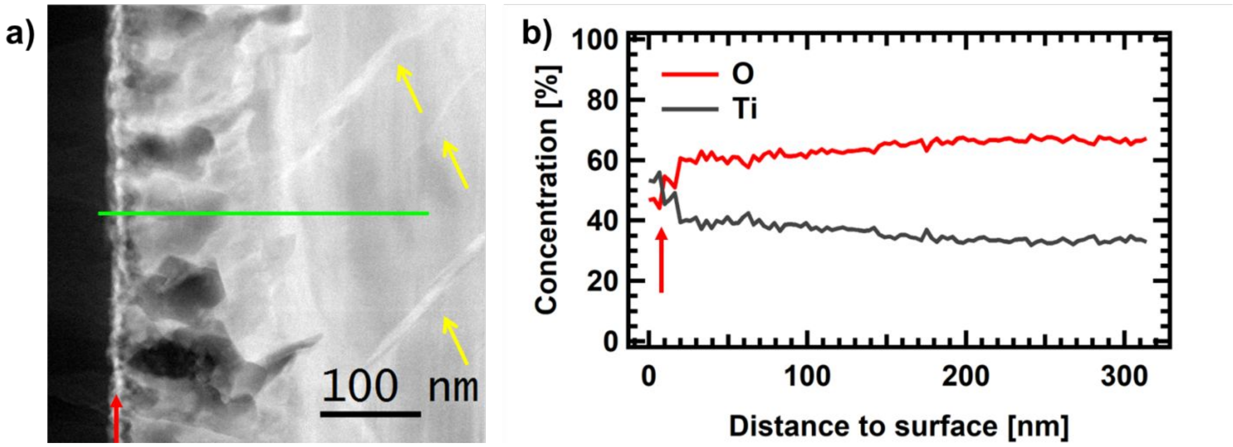

Figure S8.- a) High angle annular dark-field STEM image of the cross-section of a plasma sample etched at 950 K for 30 min. The red arrow indicates the bright surface region where the Ti/O ratio inverts, quantifiyed from the O K and Ti L2,3 edges. Yellow arrows indicate the preferential etching along the [001] surface direction. **b)** Relative Ti/O concentration profiles obtained from an EELS line scan acquired along the green line marked in (a).

7.- Analysis of the corrugation of clean and etched surfaces under UHV conditions.

Figure S9 shows the STM images and corresponding profiles taken along the TiO_2 surface before a) and after (b) and c) the atomic hydrogen etching. Specifically, two different etching conditions were used. In panel b), the surface has been etched at 500 K, while in panel c) TiO_2 has been annealed at 600 K during the exposure. In both cases, the etching has lasted 30 min. The profile of the clean surface before etching presents an average corrugation of approximately 0.5 Å. However, a clear increase in the surface corrugation is observed upon exposure to H at 500 K, passing from 0.5 to 2.0 Å (panel b)). This effect is even more pronounced if the temperature of the sample is raised to 600 K during the attack, with an average corrugation of 4.0 Å (panel c)). This increase in the surface corrugation indicates that at 500 K, mainly O_{br} atoms and some in-plane O and Ti atoms are removed from the surface, as the corrugation is lower than the

3.2 Å step height of the TiO_2 (110) surface. On the other hand, when the temperature is increased to 600 K, the etching is stronger and more layers of O and Ti atoms are involved, with O_b atoms desorbing as H_2O_b and remaining undercoordinated atoms diffusing into the bulk to occupy interstitial positions. In this way, the surface etching can be efficiently controlled by varying the sample temperature and H dose during the process, as shown below.

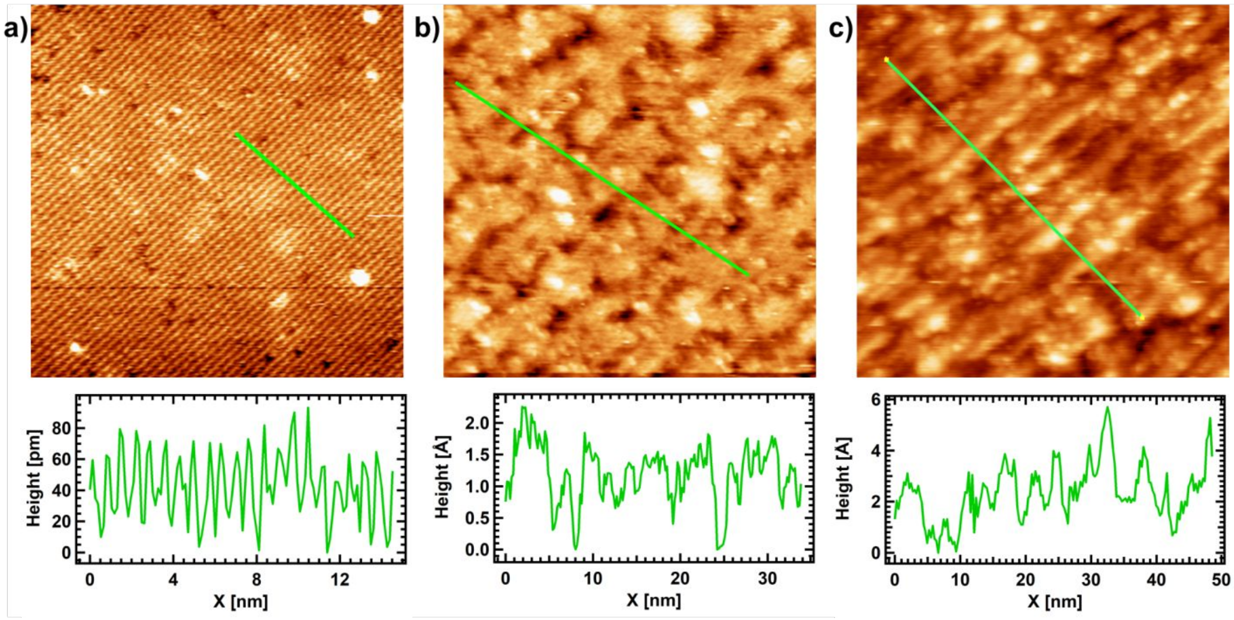

Figure S9.- STM images and corresponding STM profiles of the TiO_2 (110) surface before and after the H-etching process for two different sample temperatures. **a)** Clean surface before etching. A corrugation of 0.5 Å is observed. STM parameters: (35 nm × 35 nm), I = 36 pA, V = 1.5 V. **b)** Surface after 30 min H-etching at 500 K. STM parameters: (35 nm × 35 nm), I = 35 pA, V = 1.5 V. **c)** Surface after 30 min H-etching at 600 K. STM parameters: (50 nm × 50 nm), I = 35 pA, V = 1.5 V. Note that same experimental STM parameters were used to avoid voltage/current dependent effects.

8.- Evolution of the TiO_2 surface with atomic hydrogen dose.

Figure S10 presents a series of STM images acquired on the TiO_2 surface after six different etching times. It must be noted that in all cases both the substrate temperature and the hydrogen partial pressure at the exit of the hydrogen cracker were kept constant at 500 K and ~ 10^{-4} mbar, respectively, while the dose has been controlled by properly tuning the duration of the exposure. To obtain a more complete picture of the process, the exposure time was varied from 1 min, to study the initial etching stages, up to 30 min, to achieve a high degree of surface modification. In the low exposure regime (1 min exposure, Figure S10 a), short elongated trenches appear homogeneously distributed along the surface. The fact that there is a preference for the growth of the already available trenches instead of increasing their number indicates that the etching process

proceeds more easily in previously etched areas, most probably because those surface atoms are more exposed to the incoming atomic hydrogen and are more reactive due to their lower coordination. It is highly probable that the etching starts at defective surface sites, *i.e.* at undercoordinated surface atoms such as O_{vac}. As the exposure time is gradually increased, an increase both in the number of trenches and in their size is observed, with a concomitant decrease in the area of (1×1) surface regions (Figures S10 b-e). This is most evident after 10 min exposure (Figure S10 e), where the surface is densely covered by trenches with only small patches of the (1×1) structure that are homogeneously scattered over the surface. Finally, after 30 min exposure (Figure S10 f), the surface is completely modified and almost no (1×1) patches can be found.

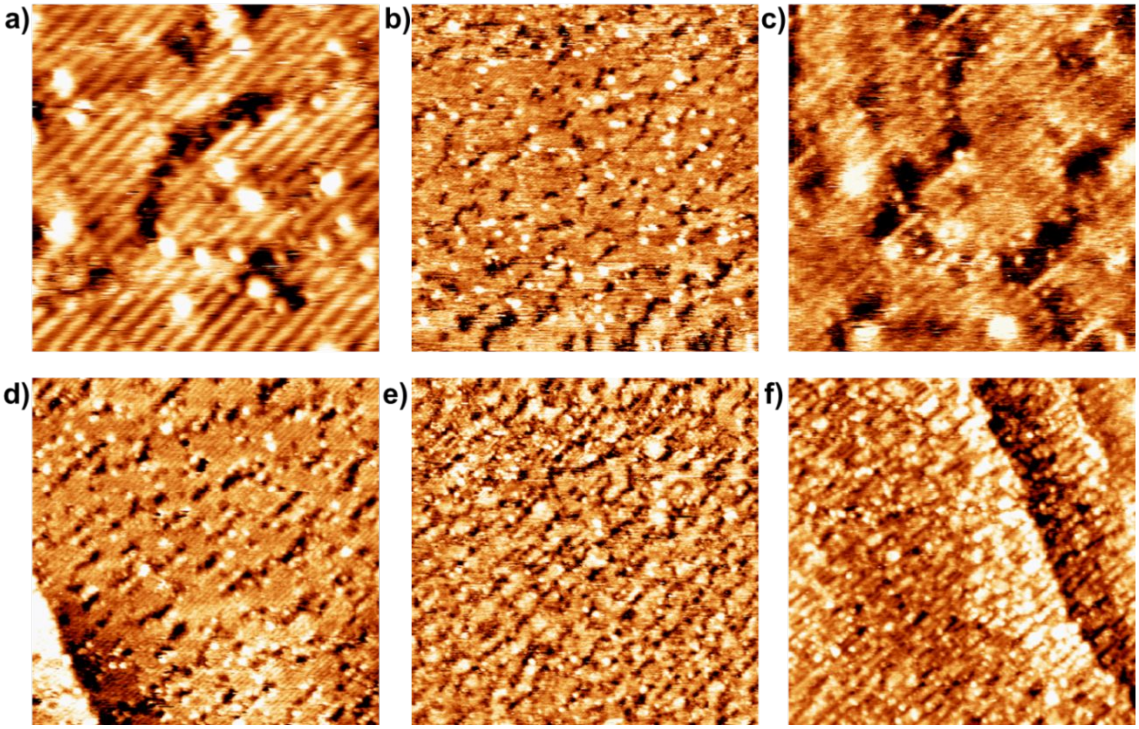

Figure S10.- Evolution of surface topography with atomic hydrogen exposure. **a)** Surface after 1 min exposure at 500 K. STM parameters: (15 nm × 15 nm), I = 52 pA, V = 1.5 V. **b)** Surface after 1.5 min exposure at 500 K. STM parameters: (75 nm × 75 nm), I = 36 pA, V = 1.5 V. **c)** Surface after 2 min exposure at 500 K. STM parameters: (15 nm × 15 nm), I = 77 pA, V = 1.5 V. **d)** Surface after 3 min exposure at 500 K. STM parameters: (50 nm × 50 nm), I = 36 pA, V = 1.5 V. **e)** Surface after 10 min exposure at 500 K. STM parameters: (75 nm × 75 nm), I = 35 pA, V = 1.5 V. **f)** Surface after 30 min exposure at 500 K. STM parameters: (75 nm × 75 nm), I = 122 pA, V = 1.5 V.

9.- Simulated STM images of the etched surface

As already introduced in Figure 3, at low hydrogen doses it is possible to discern some individual bright features located at the expected position of the Ti_{5c} atoms that we have assigned to undercoordinated Ti atoms. To further support our hypothesis, the corresponding STM images have been theoretically simulated (Figure S11). Panel ii) presents the simulated STM image for a heavily etched surface, where the O_{br} and even the in-plane O atoms have been removed. It is possible to observe the presence of bright features at the position of the undercoordinated Ti atom (blue rectangle). Additionally, the bright rows appear wider in comparison with those on the pristine surface – see simulated STM image in Fig. S11 i) – due to the lower coordination of the previously Ti_{6c} atoms – see the green rectangle in Fig. S11 ii). This is in good agreement with the experimental observations in which isolated bright features inside the trenches are observed by STM, thus further corroborating our assignation to highly undercoordinated Ti atoms (see Figure 3).

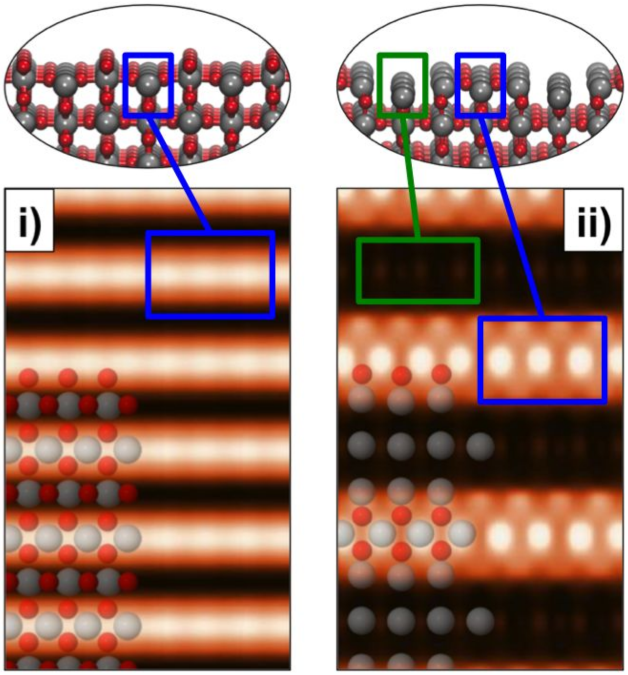

Figure S11.- Theoretical STM images (V = +1.5 V) obtained for a clean rutile TiO_2(110)-(1×1) surface (*i*), and for the reduced surface (*ii*). For the sake of clarity, side views of the surface are shown to indicate the correspondence between STM-image regions and atoms.

10.- Evolution of the TiO_2 surface with sample temperature during atomic hydrogen dose.

To experimentally confirm the existence of a non-negligible barrier in the H-induced etching of TiO_2, STM measurements after H exposure at different temperatures were performed. A summary of the results can be found in Figure S12, where a hydrogen pressure of ~ 10^{-4} mbar at the cracker exit and an exposure time of 30 min was used. If the substrate temperature is kept at 330 K, no modification of the surface structure can be observed, except for the appearance of bright patches that are associated with adsorbed H atoms (Figure S12 a). If the surface temperature is increased to 425 K, a similar situation is observed, where the (1×1) surface structure can still be distinguished under the bright, diffuse patches assigned to adsorbed H (Figure S12 b). However, some small, dark patches start becoming evident, which are attributed to the initial stages of the surface etching. If that were the case, their number should significantly increase upon a small increment of the sample temperature. Figure S12 c shows the TiO_2 surface upon exposure at 500 K. As already shown in Figure 3, the surface appears completely distorted with an average corrugation of 2 Å (Figure S9 b), thus supporting our assumption for 425 K. Finally, increasing the sample temperature to 600 K (Figure S12 d) further confirms the complete restructuration of the surface geometry and a stronger corrugation (4 Å, see Figure S9 c). Thus, these experimental findings corroborate the existence of a non-negligible energy barrier in the process with a threshold temperature in the order of 500 K.

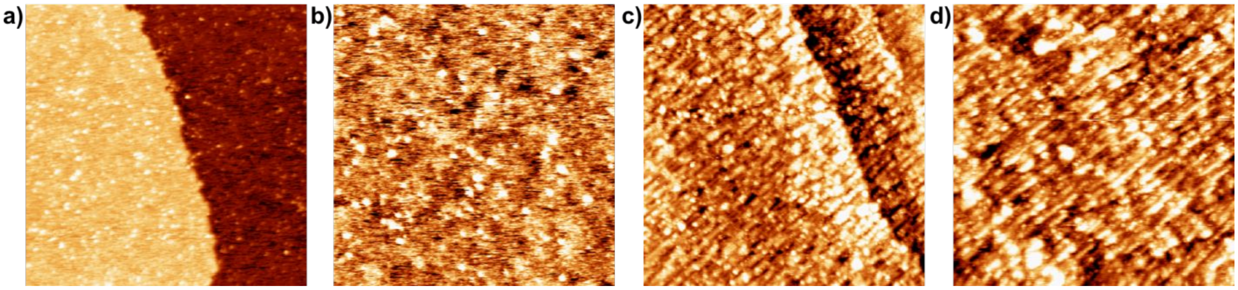

Figure S12.- Evolution of surface topography with sample temperature during etching. **a)** Exposure to H at 330 K. STM parameters: (100 nm × 100 nm), I = 35 pA, V = 1.5 V. **b)** Exposure to H at 425 K. STM parameters: (100 nm × 100 nm), I = 47 pA, V = 1.5 V. **c)** Exposure to H at 500 K. STM parameters: (100 nm × 100 nm), I = 122 pA, V = 1.5 V. **d)** Exposure to H at 615 K. STM parameters: (100 nm × 100 nm), I = 35 pA, V = 1.5 V.

11. Electrochemical Impedance Spectroscopy

Figure S13 shows the equivalent electrical circuit obtained by fitting the Nyquist plots with a Randles circuit.

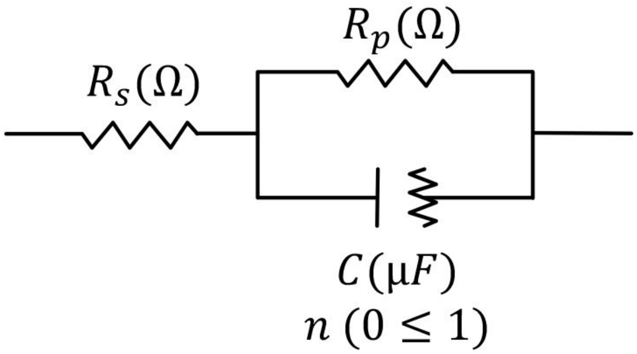

Figure S13.- Equivalent electrical circuit obtained by fitting the Nyquist plots with a Randles circuit.

The values of R_S, R_{CT}, and C_{CT} obtained are shown in Table XV.

Table XV: Resistance and Capacitance values obtained through fitting the EIS data with different equivalent circuits used

**Sample**	***S7***		***S5***		***S4***		***S3***		***S1***	
	**Dark**	**Light**	**Dark**	**Light**	**Dark**	**Light**	**Dark**	**Light**	**Dark**	**Light**
Rs / ±5Ω	101	100	75	80	81	65	70	60	80	90
Cbulk / F	2.17E-06	1.84E-06	3.50E-06	6.70E-07	5.95E-06	5.87E-06	4.20E-05	2.28E-05	4.21E-05	1.53E-05
Rbulk/ ±50Ω	14216	5380	13070	2519	20100	2609	37800	4135	119000	36080

12. Theoretical methods and models.

For the *ab-initio* structural optimizations and theoretical STM-imaging simulations of the clean and reduced TiO_2 (110) surfaces, Density Functional Theory (DFT) was used effectively combining the plane-wave and the localized-basis-set schemes as implemented in the QUANTUM ESPRESSO[6] and FIREBALL[7] simulation packages, respectively.

For the plane-wave code QUANTUM ESPRESSO,[6] one-electron wave-functions were expanded in a basis of plane-waves, with energy cut-offs of 450 and 550 eV for the kinetic energy and for the electronic density, respectively, which were adjusted to achieve sufficient accuracy in the total energy. The exchange-correlation (XC) effects were accounted by using the generalized-gradient PBE parametrization[8] and ultrasoft pseudopotentials[9] were considered to model the ion-electron interaction. In the calculations,

Brillouin zones (BZ) were sampled by means of an optimal [8×4×1] Monkhorst-Pack grid, guaranteeing a full convergence in energy and electronic density.

The localized-basis-set code FIREBALL[7] is based on a local-orbital formulation of DFT, in which consistency is implemented on the orbital occupation numbers.[11,12] These orbital occupation numbers have been obtained using the orthonormal Löwdin orbitals.[7,12–14] We have used a basis set of optimized sp^3d^5 numerical atomic orbitals (NAOs) for Ti atoms and $sp^3s^*p^{3*}$ for O atoms,[15] with the following cutoff radii (in a.u.): s=6.2, p=6.7 and d=5.7 (Ti), and $s=s^*$=3.4 and $p=p^*$=3.8 (O).[16] For these calculations, we have used the local density approximation (LDA) functional,[14] while the ion–electron interaction has been modeled by means of norm-conserving scalar-relativistic pseudopotentials.[17]

Once the electronic structure has been adequately established on the basis of the geometrical surface configurations obtained from plane-waves, theoretical STM calculations have been performed to be compared with the experimental evidence. To obtain accurate STM images and tunneling currents, we have used an efficient STM theoretical simulation technique implemented in the FIREBALL code that includes a detailed description of the electronic properties of both the tip and the sample simultaneously. Using this technique, based on a combination of a Keldysh Green's function formalism and local orbital density functional theory (DFT),[10,18] we split the system into sample and tip, where the samples here are the different surfaces considered. In these calculations, we have simulated the STM images using a W-tip formed by 5 atoms (one of them at the apex) attached to an extended W(100)-crystal. Within this approach, the STM current is given by the following expression in the tunneling regime at low temperature:[10,18]

$$I = \frac{4\pi e^2}{\hbar}\int_{E_F}^{E_F + eV_S} d\omega Tr\left[T_{ts}\rho_{ss}(\omega)T_{st}\rho_{tt}(\omega - eV_S)\right],$$

where V_s is the surface voltage, E_F is the Fermi energy, ρ_{tt} and ρ_{ss} are the density of states (DOS) matrices – in the local orbital basis – associated with the tip and sample, whilst T_{ts} and T_{st} are the local orbital Hamiltonian matrices coupling tip and sample. The overlapping Hamiltonian (hoppings) is obtained by using a dimer approximation: a dimer formed by one W atom (corresponding to the tip) and another O and Ti atom (coming from the sample) is calculated for different atom–atom distances and for all the non-zero interactions, using the Keldish-Green formalism to propagate the tunneling current between both subsystems. All the theoretical STM images have been obtained at constant-current scanning conditions – as an authentic *in silico* experiment – moving the W-tip perpendicularly to the sample in each scanning

stage to search a pre-selected fix value of the tunneling current to mimic experimental procedure. Among other factors, given the "electronic flexibility" and extended nature of the *spd* and *ss*pp** optimized for Ti and O atoms, respectively, this theoretical approach has provided excellent results in recent years, as compared with experiments, in a large variety of related literature by our group in pristine, defective and reduced TiO_2 surfaces.[16,19–21]

The clean TiO_2 (110)-(1×1) surface and its reduced stage were modelled in a repeated slab geometry with: (i) a slab of five physical TiO_2(110) layers with a minimum distance >25 Å of vacuum between neighbouring cells along the axis perpendicular to the surface; as well as (ii) full periodic boundary conditions representing infinite TiO_2(110) surfaces. In all cases, the obtained size for the unit cell in the direction parallel to the surface after full lattice optimization is (5.95×13.20) $Å^2$. For the full geometry optimizations only the three bottom TiO_2 physical layers were kept fixed in the calculations, in such a way that all the non-fixed atoms were free to move up to achieve residual forces lower than 0.01 eV $Å^{-1}$ with net cell-stresses < 0.05 GPa.

Present address: † G.S-S.: *Departamento de Física de Materiales & Instituto Pluridisciplinar, Universidad Complutense de Madrid, 28040 Madrid (Spain)*

References

(1) Gordillo-Vázquez, F. J.; Gómez-Aleixandre, C.; Albella, J. M. Influence of the Excitation Frequency on CH4/H/H2 Plasmas for Diamond Film Deposition: Electron Energy Distribution Function and Atomic Hydrogen Concentration. *Plasma Sources Sci. Technol.* **2001**, *10* (1), 99. https://doi.org/10.1088/0963-0252/10/1/313.
(2) Landi, S.; Segundo, I. R.; Freitas, E.; Vasilevskiy, M.; Carneiro, J.; Tavares, C. J. Use and Misuse of the Kubelka-Munk Function to Obtain the Band Gap Energy from Diffuse Reflectance Measurements. *Solid State Communications* **2022**, *341*, 114573. https://doi.org/10.1016/j.ssc.2021.114573.
(3) Makuła, P.; Pacia, M.; Macyk, W. How To Correctly Determine the Band Gap Energy of Modified Semiconductor Photocatalysts Based on UV–Vis Spectra. *J. Phys. Chem. Lett.* **2018**, *9* (23), 6814–6817. https://doi.org/10.1021/acs.jpclett.8b02892.
(4) Rajumon, M. K.; Hegde, M. S.; Rao, C. N. R. Adsorption of Carbon Monoxide on Ni/Ti and Ni/TiO2 Surfaces Preparedin Situ in the Electron Spectrometer: A Combined UPS-XPS Study. *Catal Lett* **1988**, *1* (11), 351–359. https://doi.org/10.1007/BF00766164.
(5) Sánchez-Sánchez, C.; Garnier, M. G.; Aebi, P.; Blanco-Rey, M.; de Andres, P. L.; Martín-Gago, J. A.; López, M. F. Valence Band Electronic Structure Characterization of the Rutile TiO2 (110)-(1×2) Reconstructed Surface. *Surface Science* **2013**, *608*, 92–96. https://doi.org/10.1016/j.susc.2012.09.019.
(6) Giannozzi, P.; Baroni, S.; Bonini, N.; Calandra, M.; Car, R.; Cavazzoni, C.; Ceresoli, D.; Chiarotti, G. L.; Cococcioni, M.; Dabo, I.; Dal Corso, A.; de Gironcoli, S.; Fabris, S.; Fratesi, G.; Gebauer, R.; Gerstmann, U.; Gougoussis, C.; Kokalj, A.; Lazzeri, M.; Martin-Samos, L.; Marzari, N.; Mauri, F.; Mazzarello, R.; Paolini, S.; Pasquarello, A.; Paulatto, L.; Sbraccia, C.; Scandolo, S.; Sclauzero, G.;

Seitsonen, A. P.; Smogunov, A.; Umari, P.; Wentzcovitch, R. M. QUANTUM ESPRESSO: A Modular and Open-Source Software Project for Quantum Simulations of Materials. *Journal of Physics: Condensed Matter* **2009**, *21* (39), 395502. https://doi.org/10.1088/0953-8984/21/39/395502.
(7) Lewis, J. P.; Jelínek, P.; Ortega, J.; Demkov, A. A.; Trabada, D. G.; Haycock, B.; Wang, H.; Adams, G.; Tomfohr, J. K.; Abad, E.; Wang, H.; Drabold, D. A. Advances and Applications in the FIREBALL Ab Initio Tight-Binding Molecular-Dynamics Formalism. *physica status solidi (b)* **2011**, *248* (9), 1989–2007. https://doi.org/10.1002/pssb.201147259.
(8) Perdew, J. P.; Burke, K.; Ernzerhof, M. Generalized Gradient Approximation Made Simple. *Phys. Rev. Lett.* **1996**, *77* (18), 3865–3868. https://doi.org/10.1103/PhysRevLett.77.3865.
(9) Vanderbilt, D. Soft Self-Consistent Pseudopotentials in a Generalized Eigenvalue Formalism. *Phys. Rev. B* **1990**, *41* (11), 7892–7895. https://doi.org/10.1103/PhysRevB.41.7892.
(10) Blanco, J. M.; González, C.; Jelínek, P.; Ortega, J.; Flores, F.; Pérez, R. First-Principles Simulations of STM Images: From Tunneling to the Contact Regime. *Phys. Rev. B* **2004**, *70* (8), 085405. https://doi.org/10.1103/PhysRevB.70.085405.
(11) Demkov, A. A.; Ortega, J.; Sankey, O. F.; Grumbach, M. P. Electronic Structure Approach for Complex Silicas. *Phys. Rev. B* **1995**, *52* (3), 1618–1630. https://doi.org/10.1103/PhysRevB.52.1618.
(12) García-Vidal, F. J.; Merino, J.; Pérez, R.; Rincón, R.; Ortega, J.; Flores, F. Density-Functional Approach to LCAO Methods. *Phys. Rev. B* **1994**, *50* (15), 10537–10547. https://doi.org/10.1103/PhysRevB.50.10537.
(13) Lewis, J. P.; Glaesemann, K. R.; Voth, G. A.; Fritsch, J.; Demkov, A. A.; Ortega, J.; Sankey, O. F. Further Developments in the Local-Orbital Density-Functional-Theory Tight-Binding Method. *Phys. Rev. B* **2001**, *64* (19), 195103. https://doi.org/10.1103/PhysRevB.64.195103.
(14) Jelínek, P.; Wang, H.; Lewis, J. P.; Sankey, O. F.; Ortega, J. Multicenter Approach to the Exchange-Correlation Interactions in Ab Initio Tight-Binding Methods. *Phys. Rev. B* **2005**, *71* (23), 235101. https://doi.org/10.1103/PhysRevB.71.235101.
(15) Basanta, M. A.; Dappe, Y. J.; Jelínek, P.; Ortega, J. Optimized Atomic-like Orbitals for First-Principles Tight-Binding Molecular Dynamics. *Computational Materials Science* **2007**, *39* (4), 759–766. https://doi.org/10.1016/j.commatsci.2006.09.003.
(16) Rangan, S.; Ruggieri, C.; Bartynski, R.; Martínez, J. I.; Flores, F.; Ortega, J. Densely Packed ZnTPPs Monolayer on the Rutile TiO2(110)-(1 × 1) Surface: Adsorption Behavior and Energy Level Alignment. *J. Phys. Chem. C* **2016**, *120* (8), 4430–4437. https://doi.org/10.1021/acs.jpcc.5b12736.
(17) Fuchs, M.; Scheffler, M. Ab Initio Pseudopotentials for Electronic Structure Calculations of Poly-Atomic Systems Using Density-Functional Theory. *Computer Physics Communications* **1999**, *119* (1), 67–98. https://doi.org/10.1016/S0010-4655(98)00201-X.
(18) Blanco, J. M.; Flores, F.; Pérez, R. STM-Theory: Image Potential, Chemistry and Surface Relaxation. *Progress in Surface Science* **2006**, *81* (10), 403–443. https://doi.org/10.1016/j.progsurf.2006.07.004.
(19) Rangan, S.; Ruggieri, C.; Bartynski, R.; Martínez, J. I.; Flores, F.; Ortega, J. Adsorption Geometry and Energy Level Alignment at the PTCDA/TiO2(110) Interface. *J. Phys. Chem. B* **2018**, *122* (2), 534–542. https://doi.org/10.1021/acs.jpcb.7b04227.
(20) Otero-Irurueta, G.; Martínez, J. I.; Lovat, G.; Lanzilotto, V.; Méndez, J.; López, M. F.; Floreano, L.; Martín-Gago, J. A. Densely Packed Perylene Layers on the Rutile TiO2(110)-(1 × 1) Surface. *J. Phys. Chem. C* **2015**, *119* (14), 7809–7816. https://doi.org/10.1021/acs.jpcc.5b00851.
(21) Wang, C.; Fan, Q.; Han, Y.; Martínez, J. I.; Martín-Gago, J. A.; Wang, W.; Ju, H.; Gottfried, J. M.; Zhu, J. Metalation of Tetraphenylporphyrin with Nickel on a TiO2(110)-1 × 2 Surface. *Nanoscale* **2015**, *8* (2), 1123–1132. https://doi.org/10.1039/C5NR03134F.
